# Supplementary material for: Carboxypeptidase activity drives L,D-transpeptidase essentiality during vegetative growth and sporulation in Clostridioides difficile
Source: mBio. 2026 Oct 6;17(11):e01580-26. doi: 10.1128/mbio.01580-26 (PMC13753750; doi:10.1128/mbio.01580-26)
Supplement: Supplementary file 1 [file mbio.01580-26-s0001.pdf]

Supplemental information for

Carboxypeptidase activity drives L,D-transpeptidase essentiality during vegetative growth and sporulation in *Clostridioides difficile*

Kevin W. Bollinger[1], Ute Müh[1], Paige B. Brannen[1], David L. Popham[2], David S. Weiss[1,3], and Craig D. Ellermeier[1,3,4]

[1]Department of Microbiology and Immunology
Carver College of Medicine
University of Iowa
Iowa City, IA, USA

[2]Department of Biological Sciences,
Virginia Tech,
Blacksburg, VA, USA

[3]Graduate Program in Genetics,
University of Iowa,
Iowa City, IA USA

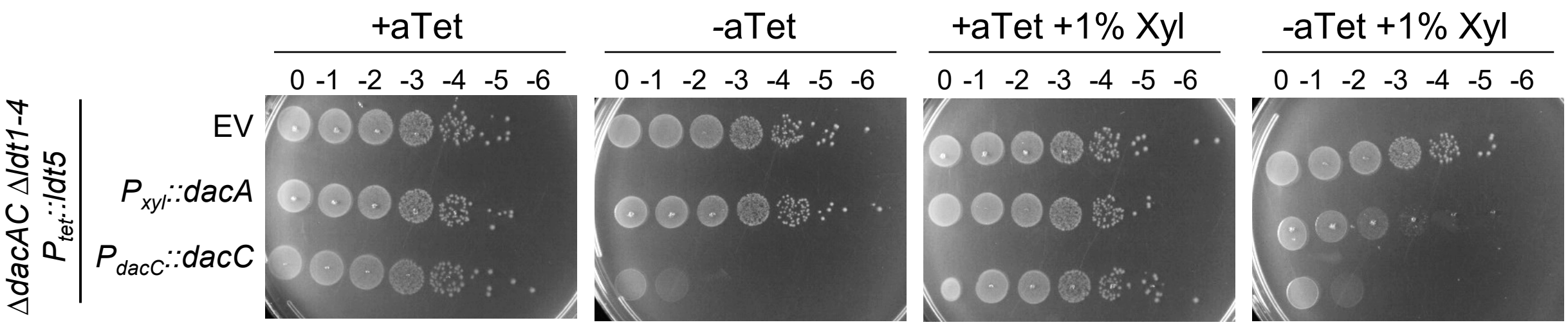

FIG S1: Complementation of DD-carboxypeptidase gene deletions in the LDT depletion strain. Spot titer assays of the LDT depletion strain harboring empty vector (EV) or plasmids that express *dacA* or *dacC*. Tenfold serial dilutions of the indicated strains were spotted onto TY Thi_{10} plates with or without 25 ng/mL aTet and 1% xylose. Plates were photographed after incubation for 20 h. Images are representative of at least three experiments.

A

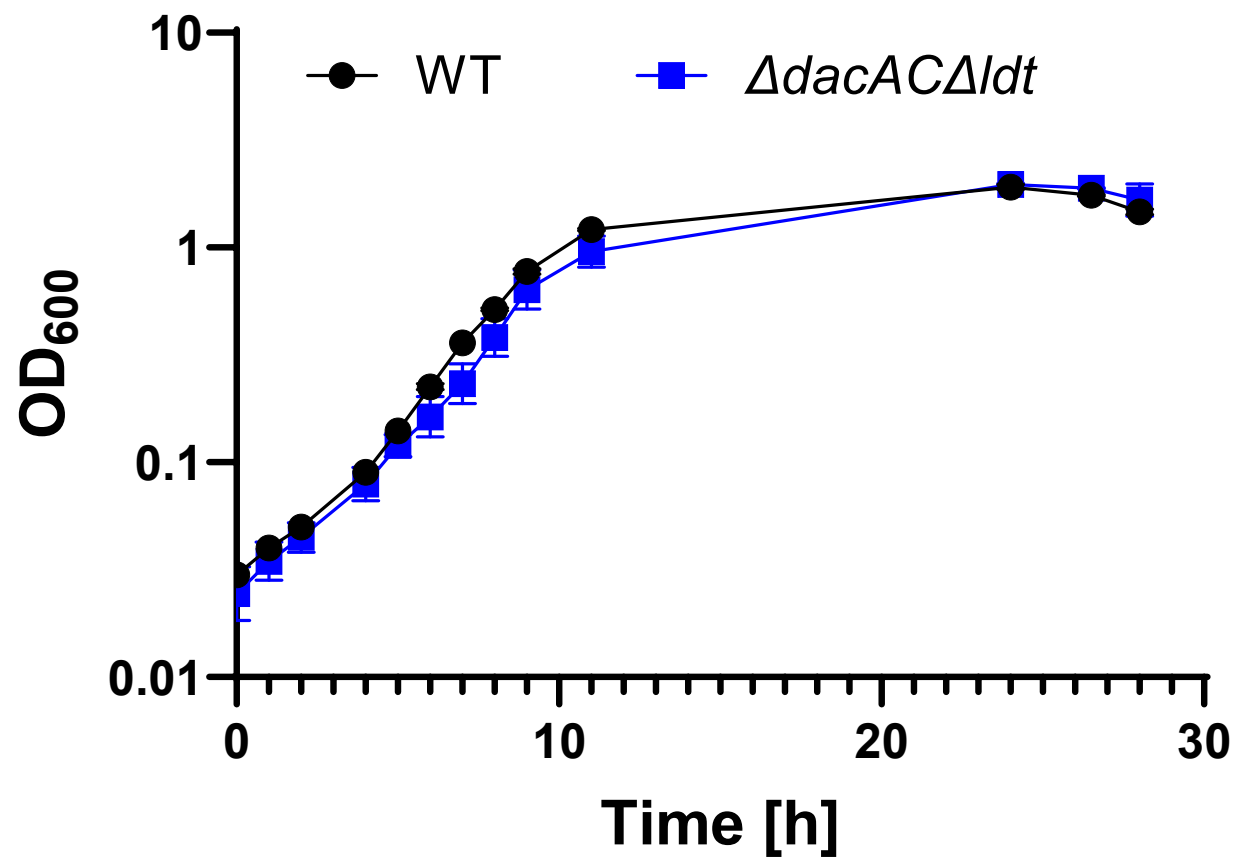

B

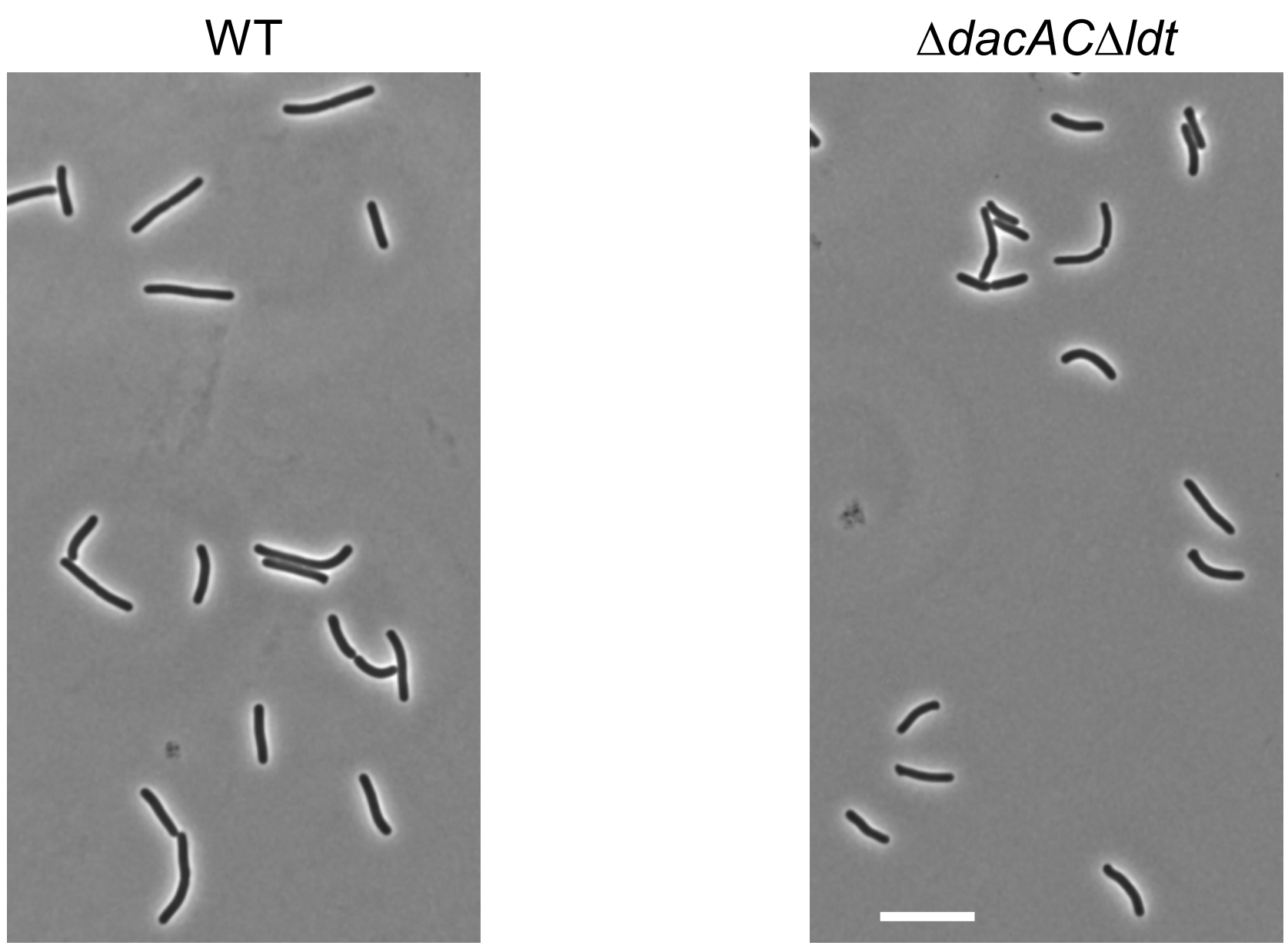

FIG S2: Δ*dacAC*Δ*ldt* grows similar to WT in minimal media with only a mild morphological defects. (A) Growth of WT and Δ*dacAC*Δ*ldt* in minimal media over the course of 30 hours. (B) Phase contrast micrographs of WT and Δ*dacAC*Δ*ldt* grown in minimal media to an OD_{600} ~0.5. Data shown are representative of three biological replicates. Scale bar = 10 µm.

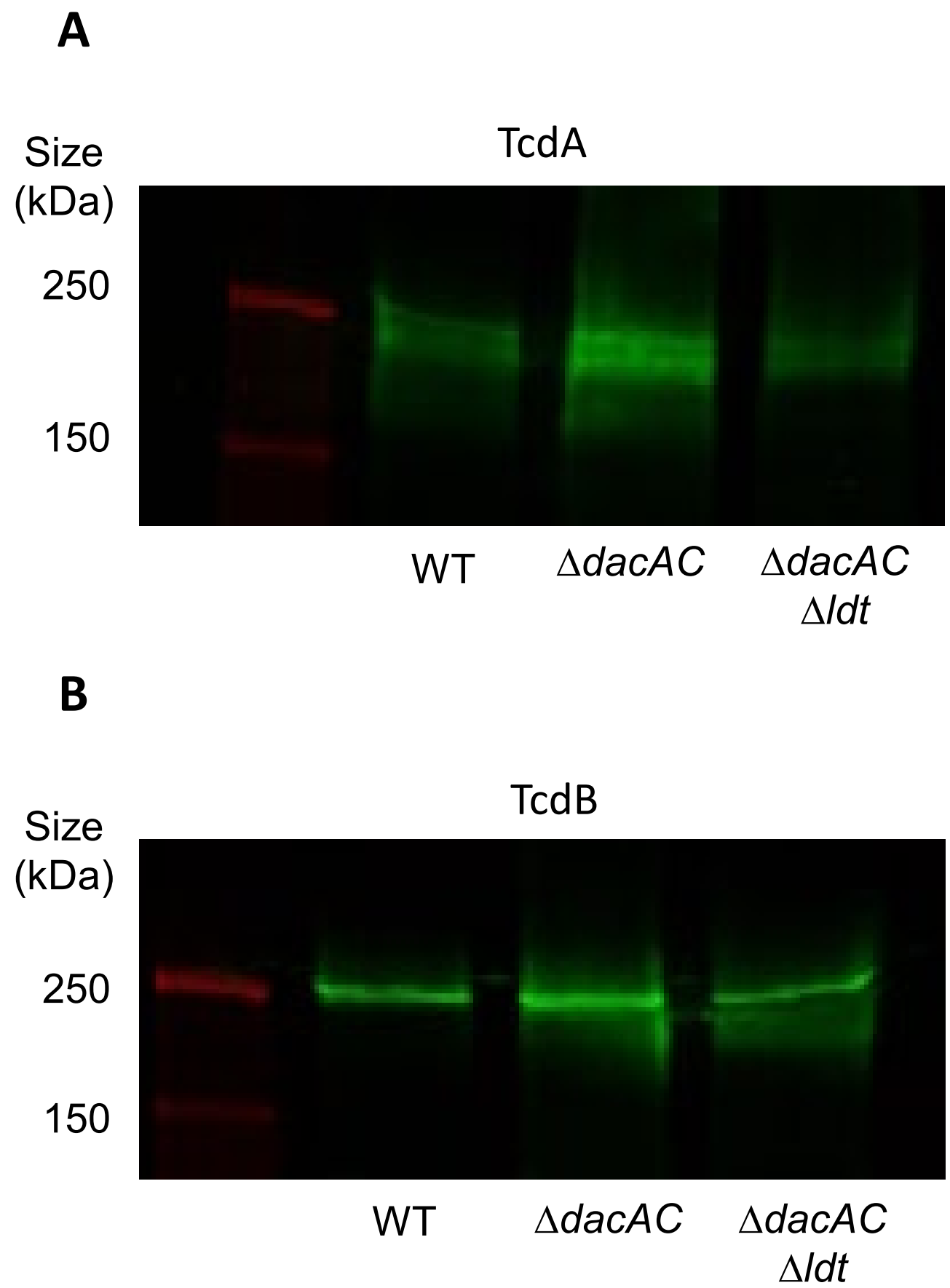

FIG S3: TcdA and TcdB secretion is not decreased in either Δ*dacAC*Δ*ldt* or Δ*dacAC*. Western blots using (A) TcdA primary antibody or using (B) TcdB primary antibody on concentrated spent media from cultures of the indicated strains after 24 h of growth. All experiments were done in biological triplicate.

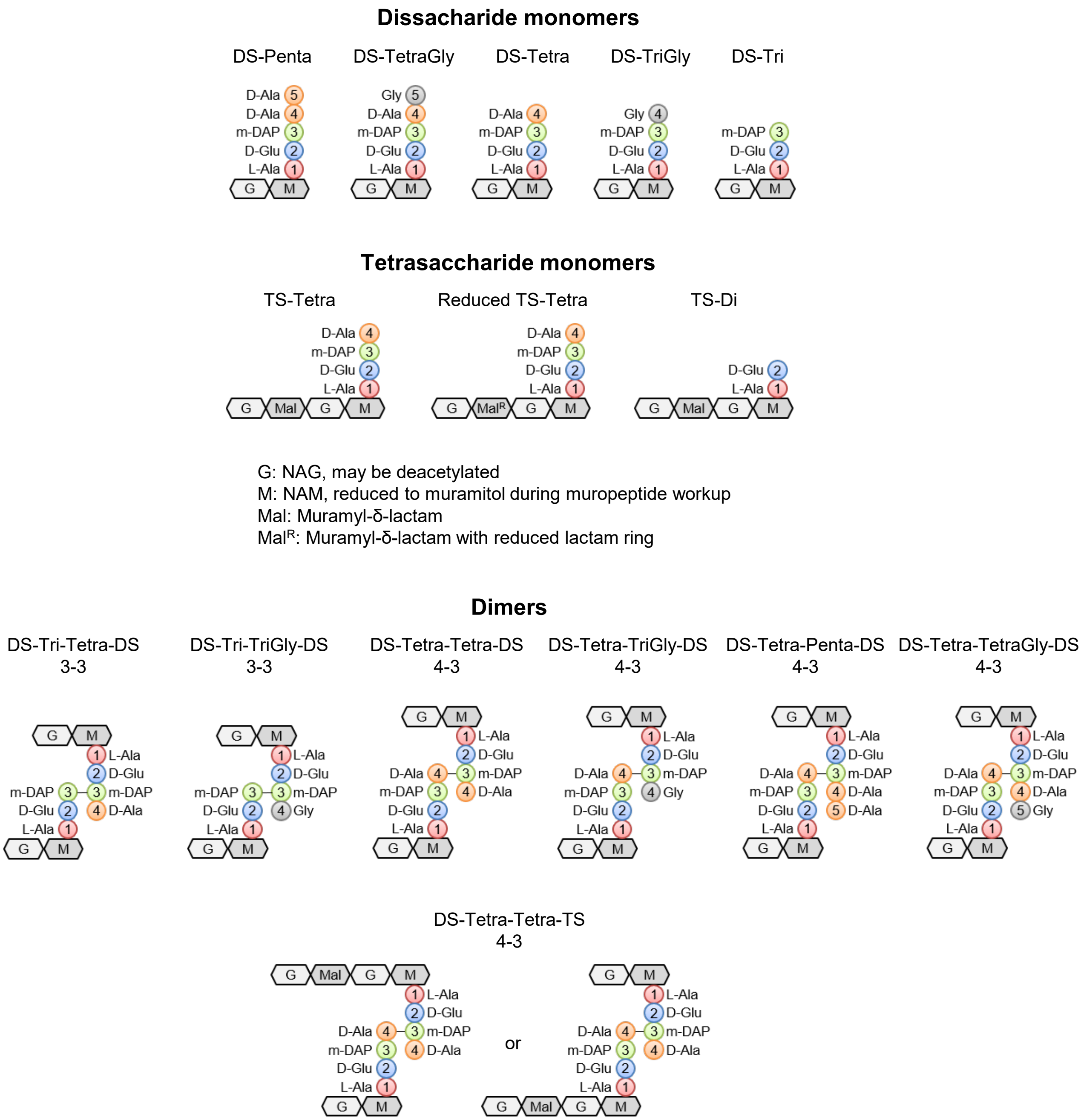

FIG S4: Schematic representation of muropeptide monomers and dimers.

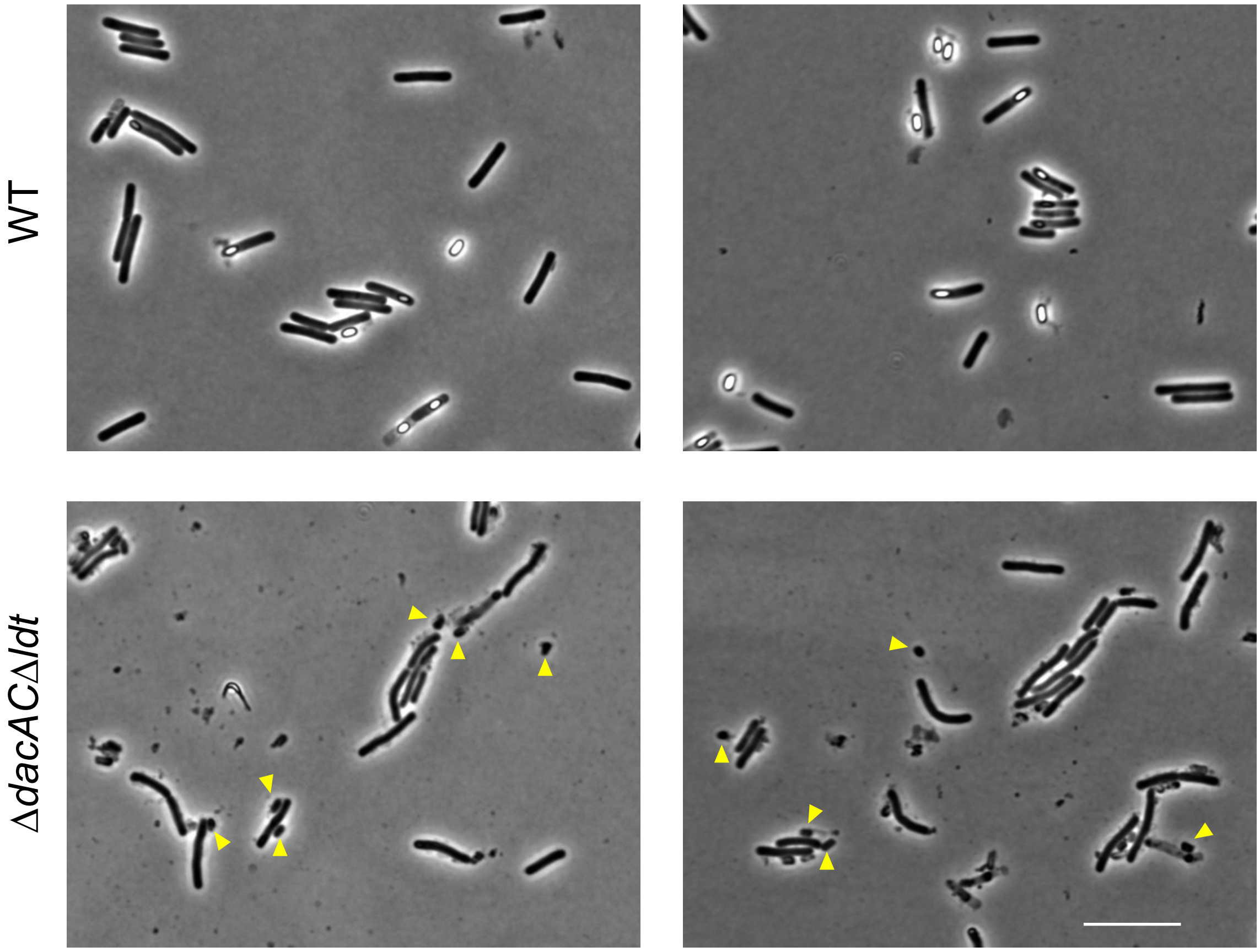

FIG S5: Additional fields of view of cells from sporulation plates related to Fig. 6C. Shown are phase contrast micrographs from two biological replicates of WT and Δ*dacAC*Δ*ldt* after 24 hours on 70:30 plates. Yellow carets indicate phase dark bodies only found in Δ*dacAC*Δ*ldt* that are inferred to be immature spores. Scale bar = 10μm

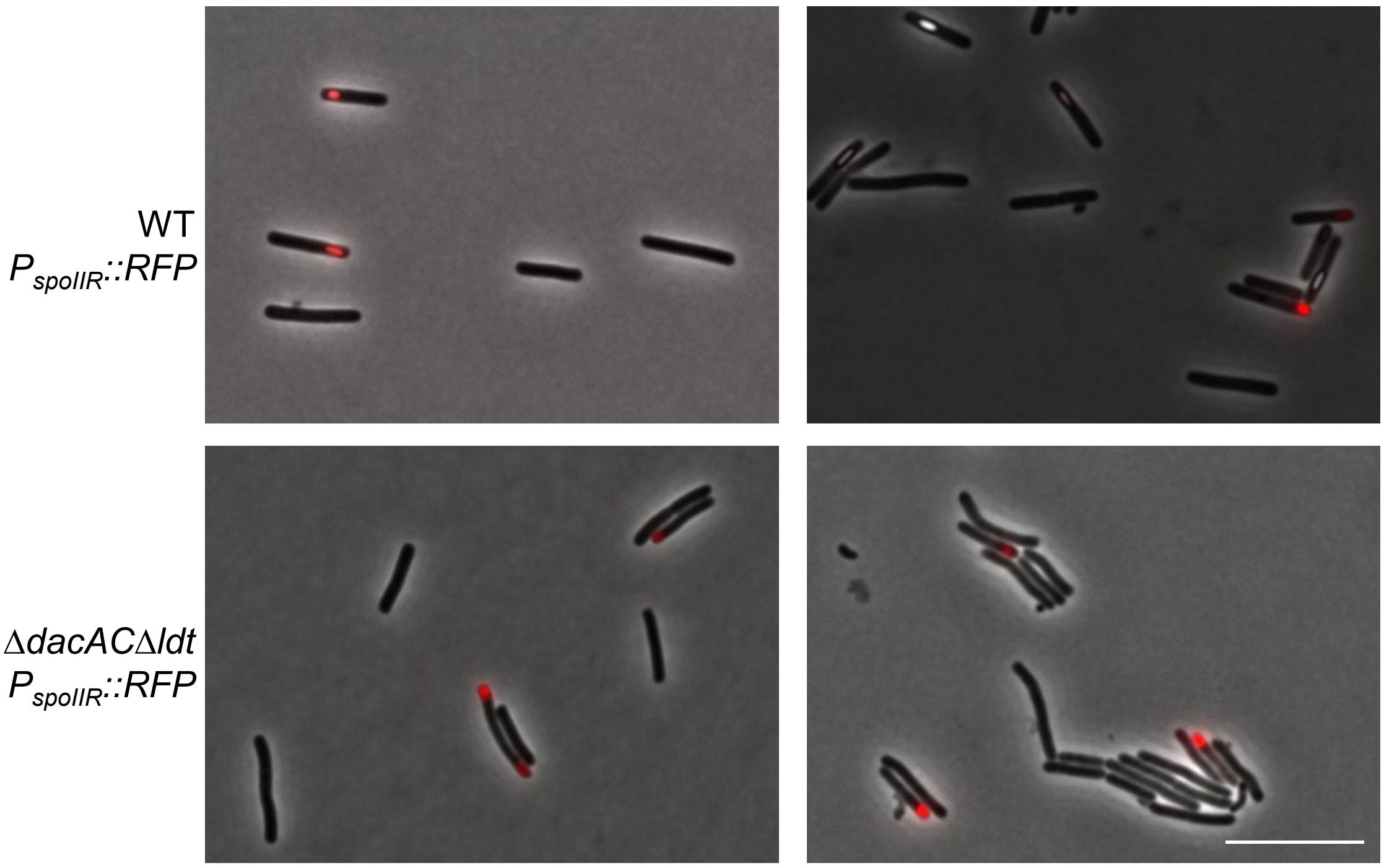

FIG S6: Additional fields of view of cells from sporulation plates related to Fig. 7. Shown are overlays of phase contrast and fluorescence images from two biological replicates. Strains are WT and Δ*dacAC*Δ*ldt* harboring a P_{spoIIR}::*rfp* reporter plasmid. Expression of *rfp* is induced during sporulation under control of σ^F. Cells were imaged after 20 hours on 70:30 sporulation plates. Scale bar = 10µm.

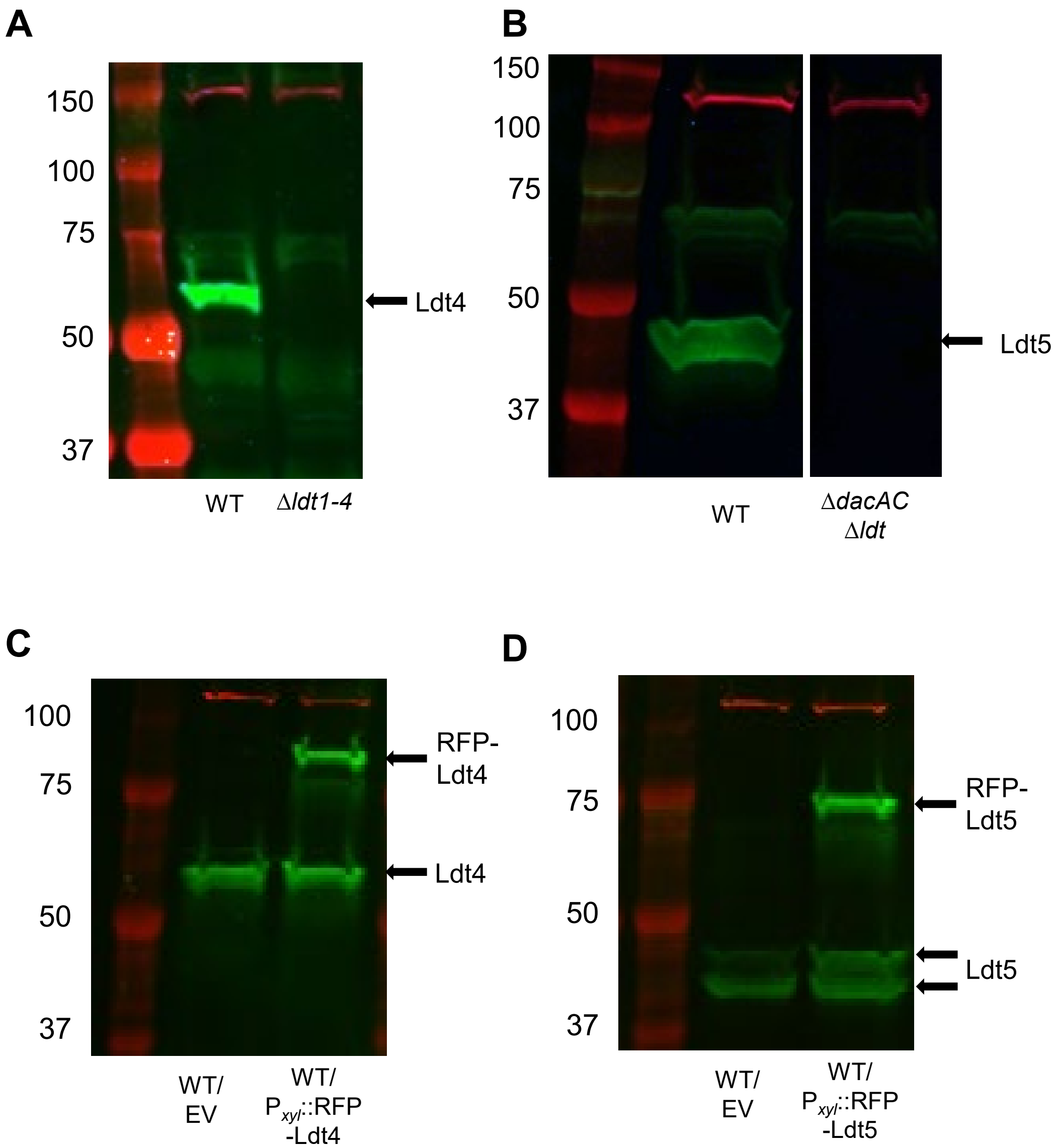

FIG S7: Western blots documenting that RFP-Ldt4 and RFP-Ldt5 are produced at physiological levels during sporulation assays. (A and B) document specificity of anti-Ldt4 and anti-Ldt5 sera because neither protein is detected in the respective null mutants. (C and D) allow comparison of the abundance of the fusions to the native Ldt proteins because the RFP fusions are produced in a WT background. To prepare samples, the indicated strains were grown for 20 hours on sporulation plates with 0.02% xylose and then harvested and processed for Western blotting as described in Methods. Numbers on the left of each blot indicate the mass of molecular weight markers, which are imaged in red. The predicted masses of the proteins of interest are: Ldt4 = 54.4 kDa, Ldt5 = 46.2 kDa, RFP-Ldt4 = 81.8 kDa and RFP-Ldt5 = 73.5 kDa. Panel B shows a single blot that was cropped to remove irrelevant lanes. Ldt5 runs as a doublet for unknown reasons.

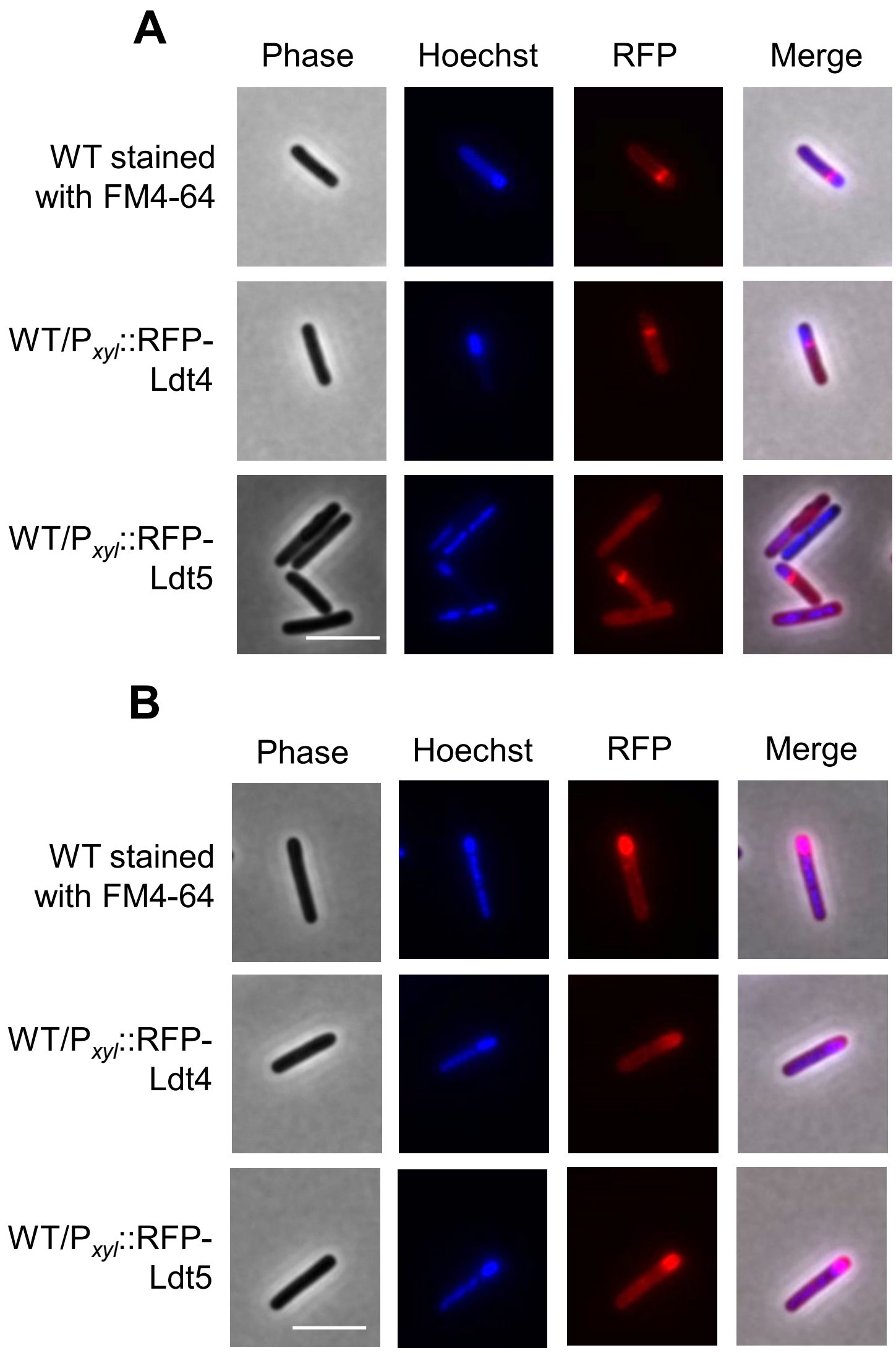

FIG S8: Ldt4 and Ldt5 localize to the asymmetric division site and around the forespore. Representative examples of cells undergoing (A) asymmetric division or (B) engulfment. WT harboring empty vector or P_{xyl}-based expression vectors for RFP-Ldt4 or RFP-Ldt5 were harvested 24 h after plating on sporulation media containing 0.02% xylose. A sample was stained with FM4-64 (red) to visualize membranes and Hoechst (blue) to visualize DNA, then photographed under phase contrast and fluorescence. A second sample was fixed and incubated overnight to allow for RFP to mature and stained with Hoechst prior to microscopy. Scale bar = 5µm.

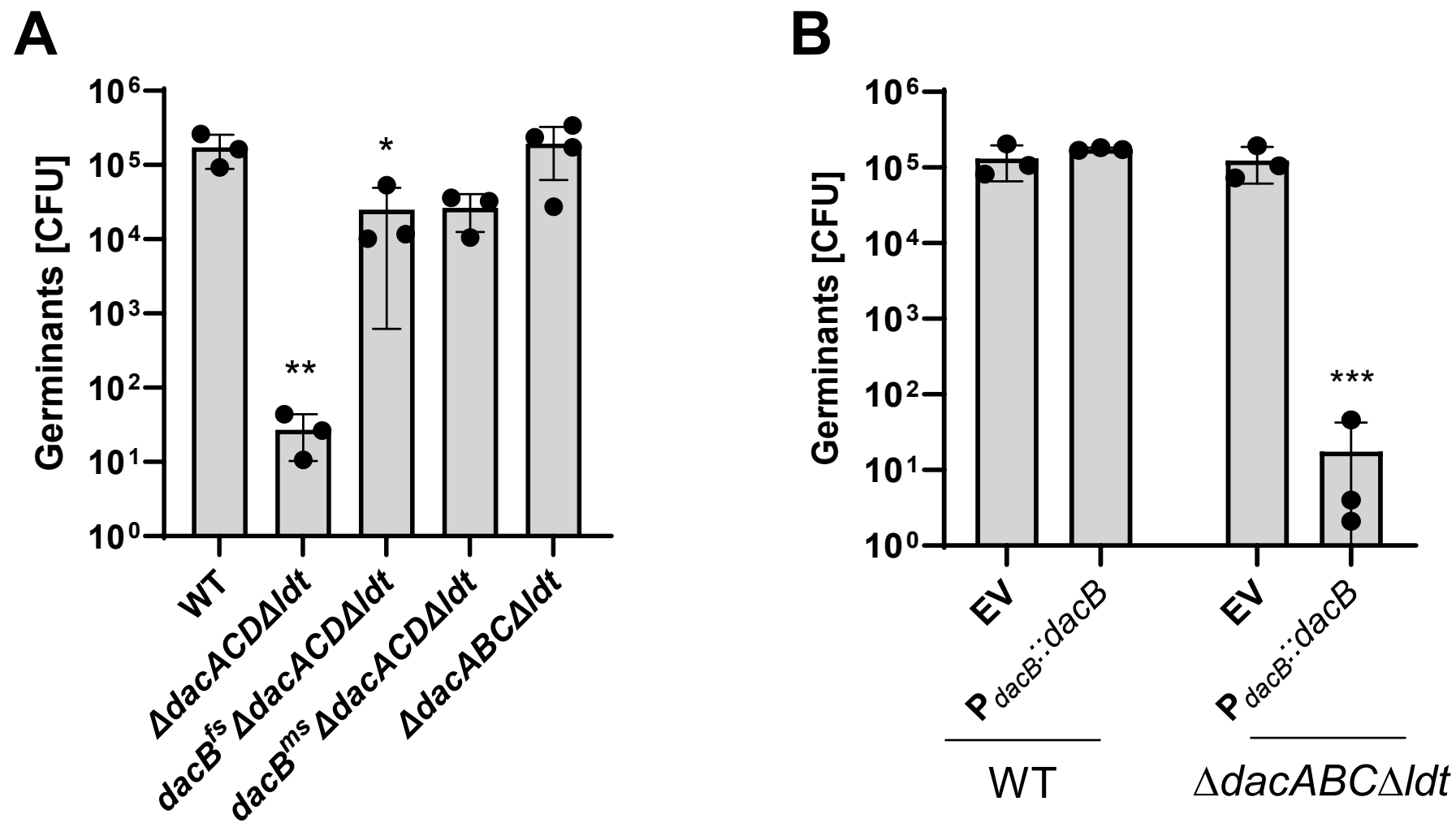

FIG S9: Loss of DacB restores sporulation in strain lacking LDTs. Sporulation by WT and the indicated mutants was quantified by determining the number of ethanol-resistant CFUs (i.e., spores) per million total CFUs (i.e, vegetative cells) 24 h after plating on sporulation media. Data graphed as the mean and standard deviation of three biological replicates. *dacB*^{fs} has a frameshift at codon 11. *dacB*^{ms} has a missense mutation at codon 209 that changes Asp to Tyr. (B) Complementation of *dacB* deletion. Note that *dacB* was expressed from its native promoter. EV = empty vector. Statistical significance was assessed using one-way ANOVA with log-transformed data followed by a Dunnett's test comparing each strain to WT or WT/EV. *=$p<0.05$; **=$p<0.01$; ***=$p<0.001$

Video S1. Live cell microscopy. Phase contrast movie of WT *C. difficile* R20291 grown on TY 1% agarose.

Video S2. Live cell microscopy. Phase contrast movie of the LDT depletion (Δ*ldt1-4* P_{tet}::*ldt5*) grown on TY 1% agarose.

Video S3. Live cell microscopy. Phase contrast movie of Δ*dacAC* grown on TY 1% agarose.

Video S4. Live cell microscopy. Phase contrast movie of Δ*dacAC*Δ*ldt* grown on TY 1% agarose.

Table S1: Plasmid details

Plasmid	Relevant features	Parent vector	Restriction enzymes to digest parent vector	PCR primers	PCR template	Assembly	Comments	Reference
pAP114	P_{xyl}::*mCherryOpt catP*						Parent plasmid for cloning under P_{xyl} control	1
pBZ101	P_{xyl} empty vector *catP*						Empty vector control for P_{xyl} induction	2
pCE938	P_{xyl}::*ldt2 catP*	pAP114	BamHI, SacI	5609+5610	R20291	ITA[1]	*cdr_2601 (ldt2)* from *C. difficile* R20291 under xylose control	3
pCE983	P_{xyl}::*ldt3 catP*	pAP114	BamHI, SacI	6243+5612	R20291	ITA	*cdr_2843 (ldt3)* from *C. difficile* R20291 under xylose control	3
pCE1175	P_{xyl}::*ldt4 catP*	pAP114	BamHI, SacI	6892+6893	R20291	ITA	*cdr_1285 (ldt4)* from *C. difficile* R20291 under xylose control	3
pCE1176	P_{xyl}::*ldt5 catP*	pAP114	BamHI, SacI	6894+6895	R20291	ITA	*cdr_2055 (ldt5)* from *C. difficile* R20291 under xylose control	3
pHL14	P_{xyl}::*mCherryOpt-ldt4 catP*							4
pHL15	P_{xyl}::*mCherryOpt-ldt5 catP*							4
pIA34	P_{xyl}::*dCas9-opt* P_{gdh}::*gRNA-neg*						CRISPRi negative control plasmid	1
pIA123	P_{xyl}::*cas9* P_{gdh}::*sgRNA-cdr_985 catP*	pCI5492	SalI, XhoI	6269+6270	pCE678	ITA	CRISPR editing parent plasmid; sgRNA-*cdr_985 (pbp2)*; no homology region	3
pKB025	P_{xyl}::*ldt1 catP*	pAP114	BamHI, SacI	5607+5608	R20291	ITA	*cdr_2797 (ldt1)* from *C. difficile* R20291 under xylose control	3
pKB092	P_{xyl}::*dacA catP*	pAP114	BamHI, SacI	7056+7057	R20291	ITA	*cdr_0441 (dacA)* from *C. difficile* R20291 under xylose control	This Study
pKB102	P_{xyl}::*cas9* P_{gdh}::*sgRNA-pgdA,* homology to delete P_{tet}::*ldt5 catP*	pIA123	PstI	6737+6738, 6739+6740	R20291	ITA	Plasmid intermediate to build P_{tet}::*ldt5* deletion plasmid	This Study
pKB112	P_{xyl}::*cas9* P_{gdh}::*sgRNA-dacC,* homology to delete *dacC catP*	pMM02	MscI, NotI	5117+4237	pIA34	ITA	CRISPR edit plasmid to delete *dacC*	This Study
pKB141	P_{xyl}::*cas9* P_{gdh}::*sgRNA-dacB,* homology to delete *dacB catP*	pKWB101	MscI, NotI	7462 + 4237	pIA34	ITA	CRISPR edit plasmid to delete *dacB*	This Study
pKB131	P_{xyl}::*cas9* P_{gdh}::*sgRNA-pgdA,* homology to delete *dacF catP*	pIA123	PstI	7394+7395, 7396+7397	R20291	ITA	Plasmid intermediate to build *dacF* deletion plasmid	This Study
pKB133	P_{xyl}::*cas9* P_{gdh}::*sgRNA-dacF,* homology to delete *dacF catP*	pKB131	MscI, NotI	7402+4237	pIA34	ITA	CRISPR edit plasmid to delete *dacF*	This Study
pKB157	P_{dacB}::dacB *catP*	pAP114	BamHI, SacI	7720+7532	R20291	ITA	*cdr_2048 (dacB)* from *C. difficile* R20291 under its native control	This Study
pKB158	P_{dacC}::dacC *catP*	pAP114	BamHI, SacI	7721+7722	R20291	ITA	*cdr_2390 (dacC)* from *C. difficile* R20291 under its native control	This Study
pKB089	P_{xyl}::*cas9* P_{gdh}::*sgRNA-dacA,* homology to delete *dacA catP*	pMM01	MscI, NotI	7040+4237	pIA34	ITA	CRISPR edit plasmid to delete *dacA*	This Study
pKWB101	P_{xyl}::*cas9* P_{gdh}::*sgRNA-pgdA,* homology to delete *dacB catP*	pIA123	PstI	7431+7432, 7433+7434	R20291	ITA	Plasmid intermediate to build dacB deletion plasmid	This Study
pMM01	P_{xyl}::*cas9* P_{gdh}::*sgRNA-pgdA,* homology to delete *dacA catP*	pIA123	PstI	6978+6979, 6980+6981	R20291	ITA	Plasmid intermediate to build *dacA* deletion plasmid	This Study
pMM02	P_{xyl}::*cas9* P_{gdh}::*sgRNA-pgdA,* homology to delete *dacC catP*	pIA123	PstI	6982+6983, 6984+6986	R20291	ITA	Plasmid intermediate to build *dacC* deletion plasmid	This Study
pRAN739	P_{spoIIR}::*mCherry*^{opt} *catP*							5

1: Isothermal assembly

Table S2: Oligonucleotides

Oligo	Sequence	Use
CDEP4237	CTTATAGGATCCGCGGCCGCTAG	Cloning
CDEP5117	AATTAAACTGTAAATGGCCA ATACTAGAGCCATCTATTTG GTTTTAGAGCTAGAAATAGC	Cloning
CDEP5607	CGATAGTTATGAAGTGAGCTGTAAGGAAGGATTTAATTATGATTGATG	Cloning
CDEP5608	TTATTAAAACTTATAGGATCCTTCGCTTAACTGTTAGCAAC	Cloning
CDEP5609	CGATAGTTATGAAGTGAGCTTAAGGAGGATGTAGTAATGTTTCTAAAAGAGGGGGA	Cloning
CDEP5610	TTTTATTAAAACTTATAGGATCCACATCTCAATTATAATTATTTTTCAATATATATTTTTACTATTTTGCA	Cloning
CDEP5612	AGTTTTATTAAAACTTATAGGATCTTGAGAATCATTATCCCAATAAAAACTC	Cloning
CDEP6243	AGATACCATAGATCCGGTACCATGAATTATAACTGTTGTTGTATCTG	Cloning
CDEP6269	AGGAGGGTAAAGAGGAGAGTCGACGCATGGATAAAAAATATAGTATAGGATTAGATATAG	Cloning
CDEP6270	CCGTATTTCTACGATGTTTTTGCTCGAGTTAATCACCACCTAATTGAGATAAA	Cloning
CDEP6737	CAGGAAGGGCGAATTCTGCAAAAACAATTAGACGAAATAATAGA	Cloning
CDEP6738	ATATTTCTCTAAAAAATTATAACTTCACCTCATTTTGACA	Cloning
CDEP6739	TGTCAAAATGAGGTGAAGTTATAATTTTTTAGAGAAATATTTGTAATAT	Cloning
CDEP6740	GAGACCGGTCAGATCTGCACACATCTATTATTTTTAGTATATAAGGA	Cloning
CDEP6811	CCCCATTATTTGCTTTTGTAATTCC	Check deletion of ldt5
CDEP6892	CGATAGTTATGAAGTGAGCTAAGGAGAGTATGGGATGAGTAATGTGAACAAA	Cloning
CDEP6893	TTATTAAAACTTATAGGATCTTAAGCTTGCGGTTGTGGTT	Cloning
CDEP6894	CGATAGTTATGAAGTGAGCTAAGGAGGTGAAGTTATGGGCAGAAGA	Cloning
CDEP6895	TTATTAAAACTTATAGGATCCTATTTTTTTAATTCTTTATAAGAACTATTTG	Cloning
CDEP6978	CAGGAAGGGCGAATTCTGCATAGAGGAATTACTAAATGGATAT	Cloning
CDEP6979	TCTTTTAATAGTATGCTTTATTCATATATTAATTAATATGCACAA	Cloning
CDEP6980	CATATTAATTAATATATGAATAAAGCATACTATTAAAAGAGTAT	Cloning
CDEP6981	CCGGTCAGATCTGCACTGCAGGGAAAGTACCTACTACAAT	Cloning
CDEP6982	CAGGAAGGGCGAATTCTGCATAACAGATGTTGCTTCACCT	Cloning
CDEP6983	TTATAATTTCCAACTACTTGTGTGAGCCTCCAAATTTTAA	Cloning
CDEP6984	TTAAAATTTGGAGGCTCACACAAGTAGTTGGAAATTATAATAAA	Cloning
CDEP6986	CCGGTCAGATCTGCACTGCAGCCAAGAAAAAAGGTTATAA	Cloning
CDEP7039	AATTAAACTGTAAATGGCCAAGCAGCACTACTTATAGACC GTTTTAGAGCTAGAAATAGC	Cloning
CDEP7040	AATTAAACTGTAAATGGCCAGGTGTTGATGGAATAAAGAC GTTTTAGAGCTAGAAATAGC	Cloning
CDEP7056	CGATAGTTATGAAGTGAGCTAAGGAGTATATGAAATGGAGGACGAGATTTTG	Cloning
CDEP7057	TTATTAAAACTTATAGGATCCTATACTTTAAAACAAACAGAATTTTTAATCT	Cloning
CDEP7058	CGTGAAGCCTTTAAGCCAACAGAAGC	Check deletion of dacA
CDEP7059	CCCTCACATCCTCAATTGTTGCC	Check deletion of dacA
CDEP7115	CACCTCTTGACTCATTCTTCTGATTTGG	Check deletion of dacC
CDEP7116	GATGCACATGGTATAGTAGGAAGTATCG	Check deletion of dacC
CDEP7282	CTTGTAGCCTTACGTTGCTCTTCAG	Check deletion of dacD
CDEP7283	GTCTCTTATTACCTTTGCGTTTGGTGC	Check deletion of dacD
CDEP7394	CAGGAAGGGCGAATTCTGCACTAAACGAAATCCAATGATACTTCCTATC	Cloning
CDEP7395	GTATTATAATTATATTTCATCTGATAATTATCGATGTCACCTCTTAAAGTTTATTTAAAC	Cloning
CDEP7396	AACTTTAAGAGGTGACATCGATAATTATCAGATGAAATATAATTATAATACACAAAG	Cloning
CDEP7397	CCGGTCAGATCTGCACTGCAGCCAGATGTAATTGACAAAACAGGTAATAGCC	Cloning
CDEP7402	AATTAAACTGTAAATGGCCAATTTTGATGGATGTTGGTAG GTTTTAGAGCTAGAAATAGC	Cloning
CDEP7431	CAGGAAGGGCGAATTCTGCAACGTTCTAATTATCCTACTTTCAGTGATTATTTAG	Cloning
CDEP7432	GTGTATTTTATACCATTTTTTGAATCTTCTTGGATTATTAAGTATATTTATATTTAAATT	Cloning
CDEP7433	TTTAAATATAAATATACTTAATAATCCAAGAAGATTCAAAAAATGGTATAAAATACAC	Cloning
CDEP7434	CCGGTCAGATCTGCACTGCAACCTAACCATCTTTCATTAACAAAGTAC	Cloning
CDEP7462	AATTAAACTGTAAATGGCCATCGTATATTGGAAGACCATT GTTTTAGAGCTAGAAATAGC	Cloning
CDEP7466	GCATTTATCAGAAGATGGTCTTATGATAG	Check deletion of dacF
CDEP7467	GGTCTTACACCAAAGCAGTATC	Check deletion of dacF
CDEP7468	GTGAGTATGAGCATGGCAAG	Check deletion of dacB
CDEP7469	CAGGCATGTCTTTTATTCCACC	Check deletion of dacB
CDEP7532	TTATTAAAACTTATAGGATCTTAAAAGTACTGAGATAATAGTTATAATTATTATCTCAG	Cloning
CDEP7650	CAATTGGATTGTGATGTAATTTGTTTGC	Check deletion of ldt5
CDEP7720	CGATAGTTATGAAGTGAGCTGTAACAGATGTTGCTTCACCTATT	Cloning
CDEP7721	CGATAGTTATGAAGTGAGCTGAAACGTTCTAATTATCCTACTTTCAGTG	Cloning
CDEP7722	TTATTAAAACTTATAGGATCCCTCCTGGTTTTATACCTCCAG	Cloning

References Supplemental Information

1. Müh U, Pannullo AG, Weiss DS, Ellermeier CD. 2019. A Xylose-Inducible Expression System and a CRISPR Interference Plasmid for Targeted Knockdown of Gene Expression in *Clostridioides difficile*. J Bacteriol 201:e00711–18.
2. Müh U, Ellermeier CD, Weiss DS. 2022. The WalRK Two-Component System Is Essential for Proper Cell Envelope Biogenesis in *Clostridioides difficile*. J Bacteriol doi:10.1128/jb.00121-22:e0012122.
3. Bollinger KW, Müh U, Ocius KL, Apostolos AJ, Pires MM, Helm RF, Popham DL, Weiss DS, Ellermeier CD. 2024. Identification of a family of peptidoglycan transpeptidases reveals that *Clostridioides difficile* requires noncanonical cross-links for viability. Proc Natl Acad Sci U S A 121:e2408540121.
4. Alberts ME, Kurtz MP, Müh U, Bernardi JP, Bollinger KW, Dobrila HA, Duncan L, Laster HM, Orea AJ, Pannullo AG, Rivera-Rosado JG, Torres FV, Ellermeier CD, Weiss DS. 2025. Analysis of essential genes in *Clostridioides difficile* by CRISPRi and Tn-seq. J Bacteriol 207:e0022025.
5. Ransom EM, Weiss DS, Ellermeier CD. 2016. Use of mCherryOpt Fluorescent Protein in *Clostridium difficile*. Methods Mol Biol 1476:53–67.
